# Stanley Brown

**Published:** 1868-04

**Authors:** 


*Obituary.*—Stanley Brown, D.D.S. died December 16, 1867, of consumption, at his residence No. 310 F Street, Washington, D. C. Dr. Brown graduated at the Baltimore College of Dental Surgery in the Class of 1866, and we learned with sincere sorrow of his, to us, sudden and unexpected death.

During his short professional career he devoted himself with untiring energy to his calling, and as an operator bid fair to acquire a high degree of excellence. During his Collegiate life he became endeared to his classmates by the many virtues that adorned his character, and his friends have our deepest sympathy for their irreparable loss.

